# Assistant Editor

**Published:** 1891-12

**Authors:** 


### ASSISTANT EDITOR.

WE have the pleasure of announcing that Joseph Head, M.D., D.D.S., has accepted the position of Assistant Editor of the INTERNATIONAL DENTAL JOURNAL, and will assume the duties in the January number. Dr. Head is well known in Eastern dental circles, and we feel that his wide experience in professional and literary work will add additional strength to the journal, and relieve the pressure of editorial responsibility.

